# Social distancing during the COVID-19 pandemic: quantifying the practice in Michigan – a “hotspot state” early in the pandemic – using a volunteer-based online survey

**DOI:** 10.1186/s12889-021-10287-w

**Published:** 2021-01-29

**Authors:** Andrea E. Cassidy-Bushrow, Mohammed Baseer, Karen Kippen, Albert M. Levin, Jia Li, Ian Loveless, Laila M. Poisson, Lonni Schultz, Ganesa Wegienka, Yueren Zhou, Christine Cole Johnson

**Affiliations:** Department of Public Health Sciences, Henry Ford Hospital, 1 Ford Place, 5C, Detroit, MI 48202 USA

**Keywords:** Social distancing, COVID-19, Pandemic, Hotspot state

## Abstract

**Background:**

Public Health policies related to social distancing efforts during the COVID-19 pandemic helped slow the infection rate. However, individual-level factors associated with social distancing are largely unknown. We sought to examine social distancing during the COVID-19 pandemic in Michigan, an infection “hotspot” state in the United States early in the pandemic.

**Methods:**

Two surveys were distributed to Michigan residents via email lists and social media following COVID-19 related state mandates in March; 45,691 adults responded to the first survey and 8512 to the second. Staying home ≥ 3 out of 5 previous days defined having more social distancing. Logistic regression models were used to examine potential factors associated with more social distancing.

**Results:**

Most respondents were women (86% in Survey 1, 87% in Survey 2). In Survey 1, 63% reported more social distancing, increasing to 78% in Survey 2. Female sex and having someone (or self) sick in the home were consistently associated with higher social distancing, while increasing age was positively associated in Survey 1 but negatively associated in Survey 2. Most respondents felt social distancing policies were important (88% in Survey 1; 91% in Survey 2).

**Conclusions:**

Michiganders responding to the surveys were both practicing and supportive of social distancing. State-level executive orders positively impacted behaviors early in the COVID-19 pandemic in Michigan. Additional supports are needed to help vulnerable populations practice social distancing, including older individuals.

## Background

**Fig. 1:**
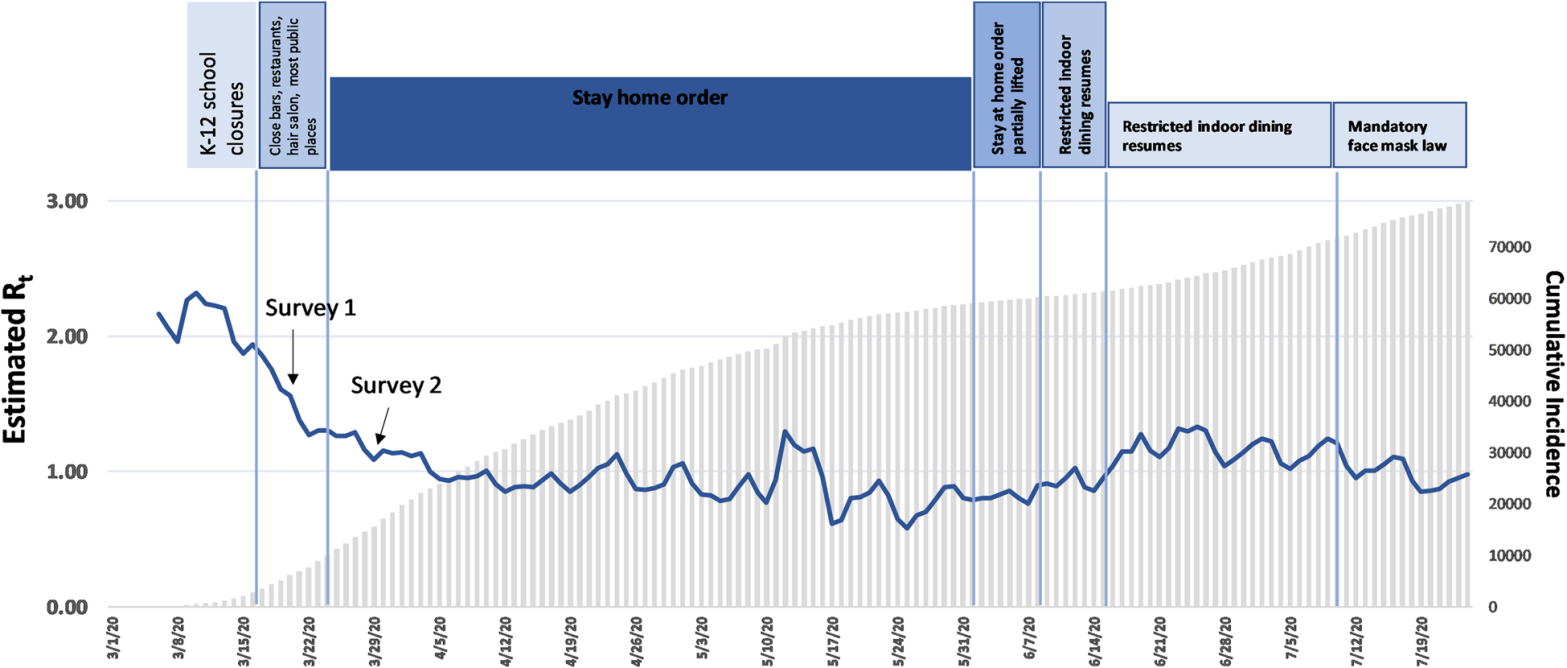
Timing of the two social distancing surveys (Survey 1 and Survey 2) against the COVID-19 social distancing policy enactments in Michigan, the cumulative incidence of COVID-19 positive cases in Michigan and the estimated effective reproduction number (R_t_). Blue shading is the confidence limits for the estimated R_t_. Dashed black line shows R_t_=1

Since the first two severe acute respiratory syndrome coronavirus 2 (SARS-CoV-2) positive cases were detected in Michigan on March 10, 2020 (Fig. 1), the state experienced growth in positive cases leading to having the seventh highest cumulative incidence of Coronavirus Disease 2019 (COVID-19) confirmed cases in the United States as of April 30, 2020. According to census estimates, Michigan had a population of over 9.9 million people in 2019, with 3.5 million living in metropolitan Detroit and 79% having household internet access [1]. The Michigan Governor implemented executive orders restricting the social interactions of residents; notably, the first major order, on March 13, 2020, closed all K-12 school buildings and banned gatherings of 250 or more people, which was followed on March 23, 2020 with the “Stay Home, Stay Safe” executive order (more closely approaching a shelter-in-place order; Fig. 1). Such orders, which were similarly implemented across the United States and other countries, were aimed at improving “social distancing” to reduce the number of physical contacts an individual has to slow the spread of an infection.

The goal of social distancing during a pandemic, such as the COVID-19 pandemic, is to reduce the effective reproduction number (R_t_) and flatten the incidence rate trajectory to reduce the number of cases burdening health care systems and reduce morbidity and mortality [2–4]. Stringent social distancing efforts during the COVID-19 pandemic have been effective in slowing the infection rate in other countries [3]. Data from the 1918 influenza pandemic reveal that factors such as public perception of disease severity, public health policies encouraging social distancing, and even weather (e.g., rain discouraging people from leaving homes) may influence social distancing practices [2]. However, little is known about the actual practice of social distancing in communities during a pandemic or the perceived importance of such practices.

Using an online-platform, email lists, and social media, two volunteer samples approximately 1 week apart, provided information on social distancing practices in Michigan during the initial phase of the outbreak in this state. The goals of this study were to (1) quantify elements of social distancing practices; (2) describe social distancing practices by geographic and demographic factors (e.g., county, age and sex of respondent); and (3) identify factors associated with greater social distancing in Michigan.

## Methods

Study data were collected and managed using REDCap (Research Electronic Data Capture), a secure, web-based software platform designed to support data capture for research studies [5, 6].

### Study population - survey 1

**Fig. 2:**
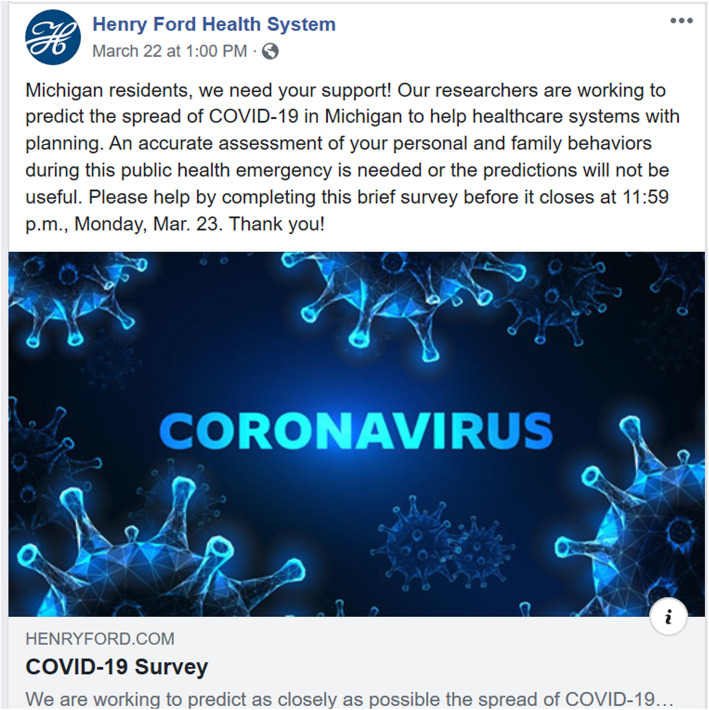
Image of the social media feed (Facebook) from Henry Ford Health System inviting residents to participate in a social distancing survey. (Note: due to overwhelming response this first survey closed early)

The first survey was live between March 21 and 22, 2020 (Fig. 1). A link to the survey was sent via email to Henry Ford Health System (HFHS) Patient and Family Advisors and community group leaders for feedback. Email recipients were encouraged to send the email to at least one other email contact. The survey was later posted on the HFHS public website and social media sites (for example, Fig. 2 shows the Facebook post). The social media link was also “shareable” and research team members, as well as the public, shared it on social media to obtain information across the state of Michigan. The survey was completely anonymous and was declared exempt from federal regulations by the HFHS Institutional Review Board (IRB).

### Study population - survey 2

The second survey was live between March 28 and 29, 2020 (Fig. 1). As before, the link was sent via email to HFHS Patient Advisors and community members. The survey was posted on the HFHS public website, but due to competing needs, not on social media. However, research team members posted the link on their own social media sites and also encouraged their contacts to do the same. The HFHS IRB declared the study exempt.

### Instrument – survey 1

Social distancing is one of the variables needed for modeling of future COVID-19 incidence rates and hospital admission surge (such as the Penn Medicine Predictive Healthcare CHIME model) [7]. The original goal of Survey 1 was to obtain a “real-world” estimate of social distancing practices in Michigan following the initial order by the Governor to close schools and limit event size. To our knowledge, no standardized questions for assessing social distancing are available, thus questions were based on hygiene practices as recommended by the Centers for Disease Control and Prevention and the White House [8], and questions from a popular online quiz (“Pandemic-Footprint”) [9]. As part of the survey introductory email and advertisement (Fig. 2), potential respondents were asked to complete a short survey (19 questions) to evaluate personal and family behaviors during the COVID-19 pandemic. The Survey 1 instrument evaluated the following:

1. *Personal and family characteristics*, including respondent age (in years), sex, number of other household members (and their ages, in years) and zip code of residence. Zip code was used to confirm residency in Michigan and to define county of residence.
2. *Health and health behaviors related to COVID-19 risk or severity*, including whether the respondent or someone in the home smokes, was currently ill with COVID-19 related symptoms (fever, cough, and shortness of breath), and whether or not they traveled in the last 14 days. Respondents were asked how often they were completing recommended pandemic hygiene (e.g., avoiding touching face and washing hands) with response options of: all of the time/I am being extra careful; most of the time/I try my best; sometimes/I do it if I think of it; or, rarely/I don’t worry about these things.
3. *Social habits,* respondents were asked about their social habits in the last 5 days, including whether they stayed home all day, had gone to their workplace or volunteer site outside the home, attended a social gathering of 10 or more people outside their home, attended a social gathering of 10 or less people outside of their home, had gone on shopping trips or outings just for fun, visited a nursing home or long-term care facility for reasons other than work, or had been in contact with someone in a COVID-19 high-risk group (e.g., someone with comorbid conditions). Responses to these questions were none of the days (0 days); a few days (1–2 days); most days (3–4 days) or every day (5 days). Respondents were asked to report if their social interactions in the last 5 days were a lot less than normal, somewhat less than normal, about the same as normal, or more than normal. Respondents were also asked to report what they thought their social behavior outside the home would be in the next 5 days as having less, about the same, more, or if they had not thought about it.
4. *Opinion on regulations*, respondents were asked what they thought of the State of Michigan shutting down restaurants, schools, and other gathering places with reporting options of it is important to do that; it could be helpful; I don’t think it will be helpful; it will do more harm than good; or I haven’t thought about it.

### Instrument – survey 2

Given the rapid change in State of Michigan orders following the first survey (March 23, 2020 “Stay Home, Stay Safe” executive order), a second survey was developed with several modifications, described below:

1. *Personal and family characteristics*, all questions were the same.
2. *Health and health behaviors related to COVID-19 risk or severity,* questions were similar to those above, with some exceptions. Questions on smoking and pandemic hygiene were removed. The question on being in contact with someone from a high-risk COVID-19 group was edited to ask if the respondent was personally considered at higher risk for COVID-19. Questions on whether or not the respondent provided care to someone outside their home and whether or not the respondent was required to leave their home for work were added. A question on handling of packages was added; participants were asked to respond by selecting one of the following options: I am taking extra steps to disinfect the packaging; I am washing my hands more, but not disinfecting the packages; I no longer order things to be delivered; I have not changed anything about how I order or handle packages; or, I never used delivery and still do not.
3. *Social habits,* questions were similar to those in Survey 1, with some changes due to the change in executive orders by the state government. The questions on attending a social gathering of 10 or more people, visiting a nursing home or long-term care facility and going on outings for fun were removed as the behaviors become prohibited.
4. *Opinion on regulations,* this question was modified slightly, with respondents being asked what do you think of the State of Michigan requiring people to shelter-in-place (Stay Safe, Stay Home policy) and asked to respond with one of the following: It is important to do that; It could be helpful; I don’t think it will be helpful; It will do more harm than good; or, I haven’t thought about it.

### Social distancing

The primary definition of social distancing was based on the number of days staying home all day. Respondents staying home ≥ 3 out of 5 days were classified as having more social distancing and those staying home < 3 of 5 days were classified as having less social distancing.

### Effective reproduction number (R_t_)

One of the main goals of social distancing is to reduce R_t_. Publicly-available cumulative and daily incidence data on COVID-19 positive cases in Michigan were obtained and used to estimate R_t_ through April 25, 2020.

### Statistical analysis

Basic descriptive characteristics including frequencies for categorical variables and means and standard deviations for continuous variables were calculated. Univariable and multivariable logistic regression models were fit to examine potential characteristics associated with higher social distancing (staying home all day ≥ 3 of the past 5 days compared to < 3 of the past 5 days). Respondent characteristics such as those generally associated with health-related behaviors (sex, age, smoking), those potentially associated with the ability to social distance (being an essential worker, number of individuals in a home, being a caregiver), and those directly part of COVID-19 social distancing policies (someone being sick in the home, recent travel) were explored.

The time-varying R_t_ was estimated using Markov Chain Monte Carlo sampling over a 3-day sliding window, by assuming that the daily incidence could be approximated by a Poisson process using the renewal equation [10]. We assumed that the mean serial interval of COVID-19 is 3.96 days with standard deviation of 4.75 [11].

## Results

### Volunteer sample – survey 1

**Fig. 3:**
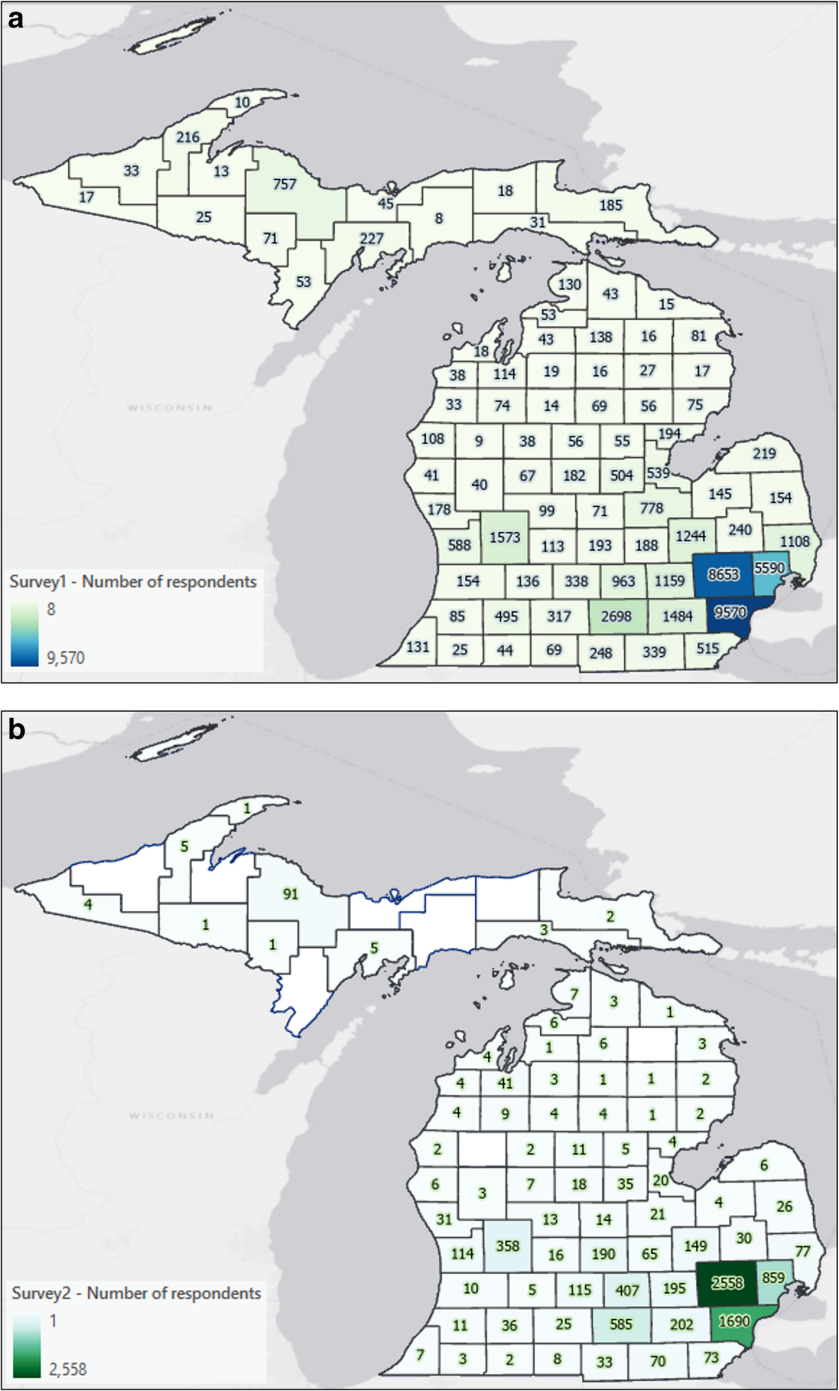
Map of Michigan showing the number of survey respondents, by county, in Survey 1 (**a**) and Survey 2 (**b**)

There were 46,974 responses between Saturday, March 21, 2020 and Sunday, March 22, 2020. Of the responses, 45,691 (97%) were from zip codes within the state of Michigan and responses were received from all 83 counties (Fig. 3a).

### Volunteer sample – survey 2

There were 8705 responses between Saturday, March 28, 2020, and Sunday, March 29, 2020. Of the responses, 8512 (97%) were from zip codes within the state of Michigan and responses were received from 75 counties in Michigan (Fig. 3b).

### Characteristics of respondents – surveys 1 and 2

In survey 1, after excluding 196 respondents due to missing sex, 230 due to missing age, and 270 due to reported age < 18 years of age, the final analytic sample size was *N*=44,995. In survey 2, after excluding 44 due to missing sex, 31 due to missing age, and 23 due to reported age < 18 years of age, the final analytic sample size was *N*=8414. Characteristics of the respondents are presented in Table 1. For both surveys, the majority of respondents were women (86% in Survey 1 and 87% in Survey 2). Respondents in Survey 1 were slightly younger (mean age 42.7 ± 12.8 years) than respondents in Survey 2 (mean age 45.9 ± 12.9 years). Similarly, the number of days people stayed home from work all 5 days increased from Survey 1 to 2 from 54 to 70%, and there was an increase in the proportion of people spending zero days in small social gatherings (< 10 people) from Survey 1 to 2 from 82 to 93%. In Survey 2, questions on factors related directly to the ability to partake in social distancing were asked; 32% of respondents reported being required to leave home for work and 8% reported they provided care for someone outside their home.

**Table 1:** Descriptive characteristics of respondents and social distancing related variables in Surveys 1 and 2 in adult respondents (> 18 years) residing in Michigan

	Survey 1 ***N***=44,995 N (%) or mean±SD	Survey 2 ***N***=8414 N (%) or mean±SD
Sex		
*Female*	38,618 (86%)	7305 (87%)
*Male*	6377 (14%)	1109 (13%)
Age (years)	42.7**±**12.8	45.9**±**12.9
Number of people in home^a^	2.3**±**1.4	2.3**±**1.5
Smoking in Home (self or others in home)^a^		
*No*	37,084 (83%)	n/a
*Yes*	7831 (17%)	n/a
Currently feeling sick with COVID-19 primary symptoms (self or others in home)^a,b^		
*No*	39,650 (88%)	7717 (92%)
*Yes*	5286 (12%)	684 (8%)
Traveled out of state in the last 14 days (self or others in home)^a^		
*No*	39,332 (88%)	7907 (94%)
*Yes*	5600 (12%)	493 (6%)
Provide care for someone who resides outside their home		
*No*	n/a	7715 (92%)
*Yes*	n/a	692 (8%)
Required to leave home for work		
*No*	n/a	5718 (68%)
*Yes*	n/a	2673 (32%)
Stayed at home all day in the last 5 days		
*Zero days*	5038 (11%)	684 (8%)
*1–2 days*	11,623 (26%)	1156 (14%)
*3–4 days*	19,796 (44%)	2582 (31%)
*5 days*	8469 (19%)	3915 (47%)
Gone to workplace or volunteer site in last 5 days		
*Zero days*	24,198 (54%)	5863 (70%)
*1–2 days*	8092 (18%)	1049 (13%)
*3–4 days*	8597 (19%)	846 (10%)
*5 days*	4052 (9%)	563 (7%)
Social gatherings > 10 people in the last 5 days		
*Zero days*	43,196 (96%)	n/a
*1–2 days*	1328 (3%)	n/a
*3–4 days*	258 (1%)	n/a
*5 days*	160 (< 1%)	n/a
Social gatherings < 10 people in the last 5 days		
*Zero days*	36,970 (82%)	7698 (93%)
*1–2 days*	7038 (16%)	384 (5%)
*3–4 days*	654 (1%)	45 (< 0%)
*5 days*	282 (1%)	136 (2%)
Outings for fun in the last 5 days		
*Zero days*	40,021 (89%)	n/a
*1–2 days*	4455 (10%)	n/a
*3–4 days*	379 (1%)	n/a
*5 days*	94 (0%)	n/a
Non-work visit to nursing home in the last 5 days		
*Zero days*	44,636 (99%)	n/a
*1–2 days*	182 (< 1%)	n/a
*3–4 days*	70 (< 1%)	n/a
*5 days*	50 (< 1%)	n/a
Contact with a person in a high risk COVID group in the last 5 days		
*Zero days*	24,879 (55%)	n/a
*1–2 days*	9077 (20%)	n/a
*3–4 days*	3315 (7%)	n/a
*5 days*	7665 (17%)	n/a
Recommended hand hygiene in the last 5 days		
*All of the time*	30,440 (68%)	n/a
*Most of the time*	13,811 (31%)	n/a
*Sometimes*	652 (1%)	n/a
*Rarely*	67 (< 1%)	n/a
Handling packages/deliveries		
*Extra steps to disinfect*	n/a	4025 (48%)
*Not disinfecting but more hand washing*	n/a	3188 (38%)
*No longer order deliveries*	n/a	400 (5%)
*No change*	n/a	328 (4%)
*Never use delivery*	n/a	465 (6%)
Social interaction in last 5 days compared to normal		
*A lot less*	38,310 (85%)	7657 (91%)
*Somewhat less*	4262 (9%)	491 (6%)
*About the same*	2099 (5%)	204 (2%)
*More*	258 (1%)	38 (< 1%)
Anticipated social interaction outside home in next 5 days		
*Less*	30,782 (68%)	4356 (52%)
*About the same*	13,347 (30%)	3786 (45%)
*More*	463 (1%)	180 (2%)
*Haven’t thought about*	361 (1%)	81 (1%)
Opinion on mandated shutdown of MI gathering places		
*Important*	39,440 (88%)	n/a
*Helpful*	4580 (10%)	n/a
*Not Helpful*	335 (1%)	n/a
*Harmful*	523 (1%)	n/a
*Don’t Know*	94 (< 1%)	n/a
Opinion on State of Michigan Shelter in Place		
*Important*	n/a	7640 (91%)
*Helpful*	n/a	616 (7%)
*Not Helpful*	n/a	60 (1%)
*Harmful*	n/a	73 (1%)
*Don’t Know*	n/a	19 (< 1%)

*COVID-19* coronavirus disease 2019, *n/a* not asked in that particular survey

^a^More than one respondent from the same household is possible

^b^For Survey 2, this refers to the respondent experiencing symptoms only

### Social distancing - surveys 1 and 2

**Fig. 4:**
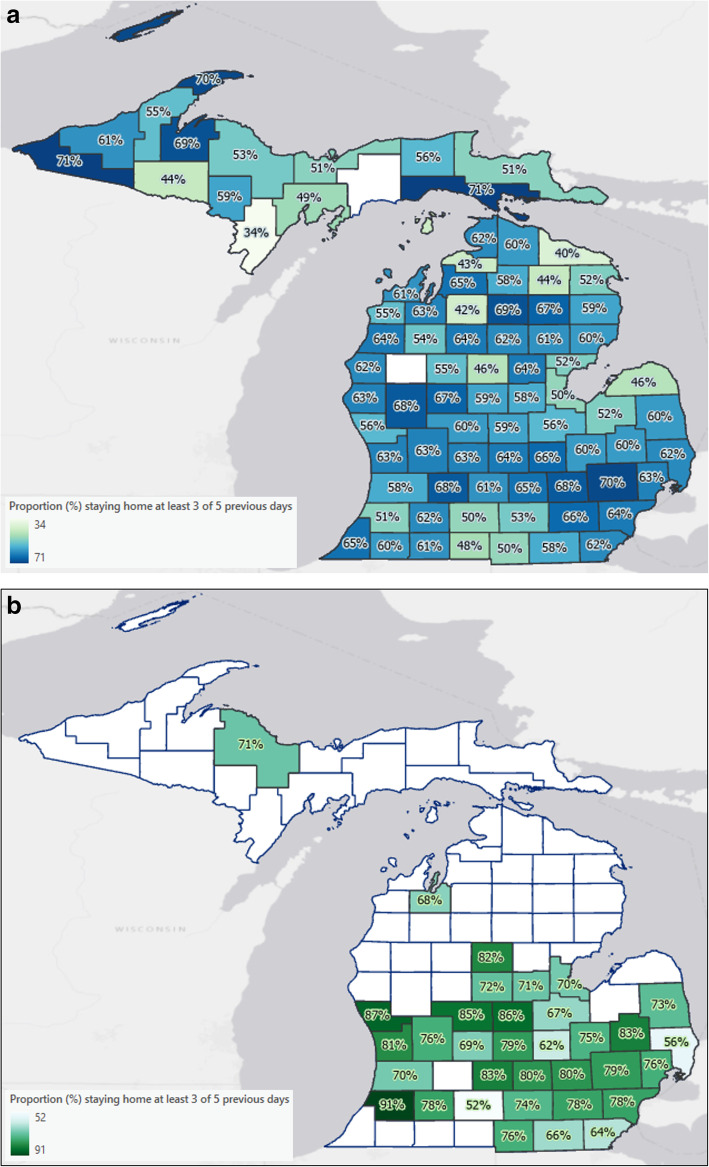
Map of Michigan showing the proportion of those reporting more social distancing (staying at home at least 3 of the 5 previous days), by county, in Survey 1 (**a**) and Survey 2 (**b**). Only counties with at least 10 recorded responses are included

In Survey 1, 63% of people reported higher social distancing (staying home at least 3 out of the 5 previous days) and this increased to 78% in Survey 2. Estimated R_t_ also decreased following the state-level executive orders for social distancing (Fig. 1). In both surveys, the proportion of respondents reporting higher social distancing varied by county of residence. Of counties with at least 10 respondents, in Survey 1 the proportion of people saying they stayed home at least 3 of the last 5 days ranged from 34 to 78% across the state (Fig. 4a) and in Survey 2 this ranged from 52 to 91% across the state (Fig. 4b).

Table 2 presents the associations of respondent characteristics with higher social distancing. For the multivariable model in Survey 1, female sex (adjusted odds ratio [aOR] = 1.74, 95% CI 1.65, 1.84), increasing age (aOR = 1.05, 95% CI 1.04, 1.06; per 5-year increase in age), and having someone (or self) sick in the home (aOR = 1.23, 95% CI 1.16, 1.31) were positively associated with higher social distancing, while smoking in the home (aOR = 0.69, 95% CI 0.65, 0.72) was the sole variable inversely associated with higher social distancing. Results were similar for Survey 2, with female sex (aOR = 1.49, 95% CI 1.27, 1.73) and the respondent being sick with COVID-19 symptoms (aOR = 1.35, 95% CI 1.09, 1.67) positively associated with higher social distancing. In contrast to Survey 1, however, increasing age (aOR = 0.95; 95% CI 0.93, 0.96; per 5-unit increase in age) was negatively associated with higher social distancing. Additionally, in Survey 2, being an essential worker was negatively associated with higher social distancing (aOR = 0.14, 95% 0.13, 0.16). Recent travel and number of people in the home were not associated with social distancing in either survey, and in Survey 2, there was no association of caregiving with social distancing (all *P* > 0.10).

**Table 2:** Univariable and multivariable associations between respondent characteristics and social distancing (number of days at home in the last 5)^a^ in adult respondents (age ≥ 18 years)

	Survey 1			Survey 2		
Variable	OR	95% CI	***P***-value	OR	95% CI	***P***-value
**Univariable analysis**						
Sex (Female vs. Male)	1.73	(1.64, 1.82)	< 0.01	1.64	(1.43, 1.88)	< 0.01
Age (years) (5-unit increment)	1.04	(1.03, 1.05)	< 0.01	0.98	(0.96, 1.00)	0.19
Smoking (self or others in home) (Yes vs. No)	0.69	(0.66, 0.73)	< 0.01	n/a	n/a	n/a
Self or someone in home sick (Yes vs. No)	1.18	(1.11, 1.25)	< 0.01	1.16	(0.95, 1.41)	0.13
Recent travel (Yes vs. No)	0.97	(0.92, 1.03)	0.36	0.89	(0.68, 1.03)	0.09
≥ 5 individuals in home (vs. < 5)	0.94	(0.82, 1.09)	0.44	1.12	(0.76, 1.63)	0.57
Essential worker (Yes vs. No)	n/a	n/a	n/a	0.15	(0.14, 0.17)	< 0.01
Caregiver (Yes vs. No)	n/a	n/a	n/a	0.81	(0.67, 0.96)	0.02
**Multivariable analysis**						
Sex (Female vs. Male)	1.74	(1.65, 1.84)	< 0.01	1.49	(1.27, 1.73)	< 0.01
Age (years) (5-year increment)	1.05	(1.04,1.06)	< 0.01	0.95	(0.93, 0.96)	< 0.01
Smoking (self or others in home) (Yes vs. No)	0.69	(0.65, 0.72)	< 0.01	n/a	n/a	n/a
Self or someone in home sick (Yes vs. No)	1.23	(1.16, 1.31)	< 0.01	1.35	(1.09, 1.67)	< 0.01
Recent travel (Yes vs. No)	0.95	(0.9, 1.01)	0.13	0.83	(0.66, 1.04)	0.10
≥ 5 individuals in home (vs. < 5)	0.90	(0.78, 1.04)	0.166	0.86	(0.57, 1.31)	0.49
Essential worker (Yes vs. No)	n/a	n/a	n/a	0.14	(0.13, 0.16)	< 0.01
Caregiver (Yes vs. No)	n/a	n/a	n/a	1.11	(0.92, 1.35)	0.29

*n/a* not asked in that particular survey, *OR* odds ratio, *CI* confidence interval

^a^Dichotomous outcome defined by “staying at home in the last 5 days” greater than or equal to 3 days

### Attitude on likelihood of social distancing and social distancing policies

The majority of respondents anticipated that they would have less social interaction in the next 5 days (68% in Survey 1 and 52% in Survey 2; Table 1). In Survey 1, respondents were asked their opinion on the governmental shutdown of gathering places; most felt this was important (88%), 10% felt it was helpful, 1% felt this was not helpful and 1% felt it was harmful (Table 1). In Survey 2, a similar question was asked about the Michigan Stay Home, Stay Safe executive order, and again, the majority (91%) felt it was important, 7% felt it was helpful, 1% felt it was not helpful and 1% felt it was harmful (Table 1).

## Discussion

Our data show, that during the growth phase of the COVID-19 epidemic in Michigan, coupled with increasing social distancing policies enacted by the Michigan government (Fig. 1), the majority (63% in Survey 1, 78% in Survey 2) of Michigan residents who responded to the surveys did not leave their home most (≥ 3 days) of the previous 5 days. This is consistent with the decrease in the estimated effective reproduction number over the same time period (R_t_; Fig. 1). Importantly, most respondents felt both the initial executive order shutting down large gatherings and the subsequent Stay Home, Stay Safe order was important (88 and 91%, respectively).

Social distancing policies are one of the most effective public health tools to combat an emerging infectious disease spread when vaccinations or treatments are not yet available. Simulation studies suggests that a combination of case isolation, household quarantine and social distance of those at higher risk would be an effective intervention for COVID-19 in Great Britain and the United States [12]. Indeed, in China, there is evidence that social distancing policies helped reduce the rate of new infections of SARS-CoV-2 and COVID-19 [13]. Recent data from the Imperial College of London suggests that the public health policies by the State of Michigan were efffective in reducing the daily incidence, number of deaths, and the R_t_ in Michigan [14].

There are limited data, however, on actual social distancing behaviors during the COVID-19 pandemic. In data from the United Kingdom between March 17–18, 2020, 45.2% reported they were taking social distancing measures [15], which is lower than the rates we found in Michigan. Unacast has developed a social distancing scoreboard based on mobile phone data to extrapolate to the population level, with metrics on change in average distance traveled, change in visitation to non-essential venues and change in human encounters (e.g., the probability of 2 mobile devices being in the same place at the same time) compared with the pre-COVID time period (February 10 to March 8, 2020) [16, 17]. During the same time period as our social distancing surveys, the state of Michigan had Unacast social distancing grades of a C- (40–50% decrease in activity) and a D (25–40% decrease in activity) on March 21 and 22, 2020, respectively, and a social distancing grade of B on both March 28 and 29, 2020 (55–70% decrease in activity) [18]. Similar to our findings, which suggested better social distancing between the first and second wave of the survey, the data from Unacast similarly showed increased social distancing over this same time period.

Factors associated with social distancing are also largely unknown. Data from the United Kingdom suggested that being over age 70 years was associated with greater social distancing and never having married was associated with lower social distancing [15]. We found several factors were consistently associated with higher social distancing in both Surveys 1 and 2: female sex and having someone (or self) sick in the home. Older age was associated with higher social distancing in the first survey, but was associated with lower social distancing in the second survey. While the reasons for this are unclear, it is possible that once the executive order reduced the number of people traveling to work, older individuals, who may not be familiar with or able to access overwhelmed grocery delivery or other services, were actually leaving the home more than younger respondents. In the first survey, smoking (self or others in home) was associated with lower social distancing; however, this question was not asked in the second survey, so we were unable to compare results between the two surveys. Smoking may generally reflect an overall difference in health-related behaviors, which may explain this association. Finally, as expected, in Survey 2 (this was not asked in Survey 1), respondents who reported they were required to leave their home for work were significantly less likely to have higher social distancing. These findings suggest a need to better understand factors associated with both the ability and the desire to participate in social distancing practices.

Since anyone with the survey link was able to access the survey, and no identifying information was collected, it is not possible to know if a single person completed the survey more than once or if multiple individuals within a household completed the survey. In both cases, a lack of independence between individuals completing the survey may have led to an underestimation of the variance for the association tests applied and a concomitant overestimation of statistical significance. In the first survey, there was no collection of information about whether the respondent conducted “essential work.” Since the survey was distributed through a health system website, it is possible that a large proportion of health care employees completed the survey. Further, since the survey was voluntary and circulated freely on social media sites, we recognize that the sample is likely biased toward health-conscious respondents. The sample is primarily female and thus results may not be generalizable to men. Future studies that could be implemented in other settings to reach respondents who do not participate in social media, for example, are needed. As such, our results likely reflect the upper bound of social distancing practices in Michigan during the early phases of the COVID-19 pandemic. However, at times when social distancing is required, “virtual research” is the only option available and methodologies to optimize these approaches is warranted.

## Conclusions

In summary, the Executive Orders in Michigan to implement social distancing policies were associated with both increases in social distancing behaviors (defined in the current study as staying home for at least 3 days over a defined 5 day period) and with reduction in R_t_. However, additional supports may be needed to help vulnerable populations, such as older individuals, practice social distancing. Finally, additional study is needed to better understand social distancing behaviors, including the impact of loosening restrictions after COVID-19 confirmed positive case rates begin to slow.

## Data Availability

The datasets used and/or analyzed during the current study are available from the corresponding author on reasonable request.

## Abbreviations

aOR: Adjusted odds ratio
COVID-19: Coronavirus Disease 2019
HFHS: Henry Ford Health System
IRB: Institutional Review Board
REDCap: Research Electronic Data Capture
R_t_: Effective reproduction number
SARS-CoV-2: Severe acute respiratory syndrome coronavirus 2
